# Microfibre-Functionalised Silk Hydrogels

**DOI:** 10.3390/cells13010010

**Published:** 2023-12-20

**Authors:** Jirada Kaewchuchuen, Napaporn Roamcharern, Suttinee Phuagkhaopong, Luis M. Bimbo, F. Philipp Seib

**Affiliations:** 1 Strathclyde Institute of Pharmacy and Biomedical Sciences, University of Strathclyde, 161 Cathedral Street, Glasgow G4 0RE, UKluis.bimbo@ff.uc.pt (L.M.B.); 2 Department of Pharmacology, Faculty of Medicine, Chulalongkorn University, Bangkok 10330, Thailand; 3 Department of Pharmaceutical Technology, Faculty of Pharmacy, University of Coimbra, 3000-548 Coimbra, Portugal; 4 CNC—Center for Neuroscience and Cell Biology, University of Coimbra, Rua Larga, 3004-504 Coimbra, Portugal; 5 CIBB—Center for Innovative Biomedicine and Biotechnology, University of Coimbra, Rua Larga, 3004-504 Coimbra, Portugal; 6 Fraunhofer Institute for Molecular Biology & Applied Ecology, Branch Bioresources, Ohlebergsweg 12, 35392 Giessen, Germany; 7 Institute of Pharmacy, Friedrich Schiller University Jena, Lessingstr. 8, 07743 Jena, Germany

**Keywords:** silk fibroin, fibre, mechanics, stem cells, gel, tissue engineering, iPSC

## Abstract

Silk hydrogels have shown potential for tissue engineering applications, but several gaps and challenges, such as a restricted ability to form hydrogels with tuned mechanics and structural features, still limit their utilisation. Here, *Bombyx mori* and *Antheraea mylitta* (Tasar) silk microfibres were embedded within self-assembling *B. mori* silk hydrogels to modify the bulk hydrogel mechanical properties. This approach is particularly attractive because it creates structured silk hydrogels. First, *B. mori* and Tasar microfibres were prepared with lengths between 250 and 500 μm. Secondary structure analyses showed high beta-sheet contents of 61% and 63% for *B. mori* and Tasar microfibres, respectively. Mixing either microfibre type, at either 2% or 10% (*w*/*v*) concentrations, into 3% (*w*/*v*) silk solutions during the solution–gel transition increased the initial stiffness of the resulting silk hydrogels, with the 10% (*w*/*v*) addition giving a greater increase. Microfibre addition also altered hydrogel stress relaxation, with the fastest stress relaxation observed with a rank order of 2% (*w*/*v*) > 10% (*w*/*v*) > unmodified hydrogels for either fibre type, although *B. mori* fibres showed a greater effect. The resulting data sets are interesting because they suggest that the presence of microfibres provided potential ‘flow points’ within these hydrogels. Assessment of the biological responses by monitoring cell attachment onto these two-dimensional hydrogel substrates revealed greater numbers of human induced pluripotent stem cell-derived mesenchymal stem cells (iPSC-MSCs) attached to the hydrogels containing 10% (*w*/*v*) *B. mori* microfibres as well as 2% (*w*/*v*) and 10% (*w*/*v*) Tasar microfibres at 24 h after seeding. Cytoskeleton staining revealed a more elongated and stretched morphology for the cells growing on hydrogels containing Tasar microfibres. Overall, these findings illustrate that hydrogel stiffness, stress relaxation and the iPSC-MSC responses towards silk hydrogels can be tuned using microfibres.

## 1. Introduction

Tissue engineering, as a pivotal procedure in regenerative medicine, requires innovative strategies to repair and replace tissues damaged or lost after wounding [1,2]. Its success depends on the availability of biomaterials that can facilitate tissue regeneration, for example, by providing supportive hydrogels for cell attachment [3,4], proliferation [5] and differentiation [6,7]. The use of biopolymers is particularly prominent in wound care, and interesting platforms are emerging, including, for example, cellulose-based hydrogels doped with chitosan nanoparticles [8] or drug-loaded polydopamine for the treatment of skin wounds [9]. Silk fibroin, derived from *Bombyx mori* silkworms, has also garnered significant interest among tissue engineers, in part due to its biocompatibility, biodegradation and resemblance to the natural extracellular matrix (ECM) (reviewed in [10,11]). For example, silk hydrogels can mimic the viscoelastic properties of tissues [12], making them an interesting platform for further exploration of their use in cell attachment and growth as well as in the scaffold fabrication required for more advanced tissue engineering applications. 

Tuning the material mechanics of cell substrates is an integral part of tissue engineering. The silk fibre reinforcement strategy was first pioneered in tissue engineering scaffolds [13], it has since been used in polymer hydrogels to enhance their mechanical strength [6,14,15,16]. For example, reinforcement of silk hydrogels with 2% (*w*/*v*) *B. mori* fibres 500 μm in length nearly doubled its hydrogel mechanical strength [14]. However, further studies have confirmed that the incorporated microfibres do more than just enhance mechanical support; they also enhance the behaviour of the cells that attach to them [14]. This phenomenon has also been observed with other hydrogel systems reinforced with *B. mori* fibres (e.g., [6,15,16]). 

To date, *B. mori* silk has been the material of choice for silk-mediated tissue engineering for several reasons. This preference for *B. mori* silk is due, at least in part, to the robust supply chain of *B. mori* raw materials, the ease with which the fibre can be reverse-engineered into liquid silk and the clinical approval and use of *B. mori* silk in humans, both as its native spun fibre and its processed formats [11,17]. However, many different silks exist in nature; indeed, silk has arisen at least 23 times via independent evolutionary events [18]. This diversity guarantees that silk will continue to amaze. Even among Lepidoptera, the silks produced by species other than *B. mori* provide a rich library with sequence variations that can be tapped into. For example, the silk produced by *Antheraea mylitta* (the Tasar silkworm) shares many similarities with *B. mori* silk, but it differs in subtle but important ways in its primary protein sequence. For example, in contrast to *B. mori* silk fibroin, Tasar silk fibroin contains (i) a higher number of alanine residues [19] and (ii) seven arginyl-glycyl-aspartic acid (RGD) sequences per molecule, which promote cell adhesion via integrin receptor engagement [10,20]. Because *B. mori* silk lacks this RGD sequence, Tasar silk is an important inclusion in the ‘silk toolbox’ [10]. Indeed, Tasar silk films and scaffolds have been shown to significantly improve primary cardiomyocyte performance, including cell attachment and proliferation [21] (reviewed in [20]). The availability of integrin engagement for cell adhesion provided by Tasar silk is also relevant for other cell types, including induced pluripotent stem cells (iPSCs). iPSCs are particularly promising contenders in emerging tissue engineering therapies because they provide the unique opportunity of culturing limitless patient-specific stem and progenitor cells (reviewed in [22]), as well as iPSC-derived mesenchymal stem cells (MSCs). 

Human iPSC-derived MSCs (iPSC-MSCs) are particularly promising cells for tissue engineering purposes because of their potential for multilineage differentiation [23,24] and their immunomodulatory properties [25]. The ability of iPSC-MSCs to differentiate into various cell lineages [24] can greatly aid tissue repair [24], regulate immune responses and mitigate inflammation [25]. This combination of regenerative and immunomodulatory potentials provides the unique possibility of fabricating tissue engineering constructs with enhanced regenerative outcomes. Ultimately, iPSC-MSCs will benefit from the fabrication of novel delivery platforms that maximise their therapeutic potential [26].

In the present study, we created silk hydrogels reinforced with either *B. mori* or Tasar silk microfibres, and we characterised the hydrogel mechanics and assessed their cell culture performance using iPSC-MSCs. As part of this study, we also established a novel protocol for manufacturing Tasar silk microfibres. Both silk microfibre types were characterised, and each was embedded in a physically crosslinked *B. mori* silk hydrogel for the assessment of their impact on hydrogel mechanics. The modified hydrogels were then used to assess iPSC-MSC cell attachment and viability.

## 2. Materials and Methods

### 2.1. B. mori Silk Degumming and Solution Preparation

The *B. mori* silk fibroin solution was prepared as detailed previously [27]. Briefly, *B. mori* cocoons were cut and degummed in 0.02 M Na_2_CO_3_ solution at 100 °C for 60 min. The degummed silk fibres were rinsed three times with deionised water and dried in a fume hood overnight. The dried silk was dissolved in a fresh 9.6 M LiBr solution at 60 °C for 3 h. This solution was dialysed against deionised water using a dialysis cassette (molecular weight cut-off 3500 Da; Thermo Fisher Scientific Inc., Waltham, MA, USA) with six water changes over 3 days. The silk solution was collected and centrifuged for 20 min twice at 5 °C and 9500× *g*. The final silk solution was typically 5–6% (*w*/*v*) and was stored at 4 °C until use.

### 2.2. A. mylitta Silk Degumming

Tasar cocoons were obtained from Latifi Silk Export LLT (Bhagapur, India). The Tasar silk was degummed based on an earlier work [28]. Briefly, dried cocoons were cut into 5 × 5 mm pieces and 5 g were degummed with 2 L of 0.025 M Na_2_CO_3_ for 60 min, followed by 60 min in 2 L of 0.0125 mM Na_2_CO_3_. The silk fibres were then washed for 20 min three times with 1 L of distilled water and then dried in a fume hood overnight. 

### 2.3. Microfibre Production

**Figure 1:**
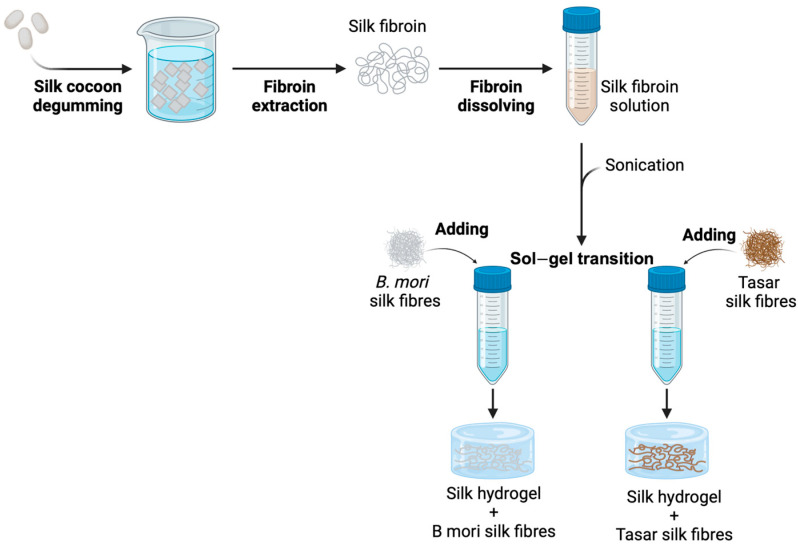
Manufacture of *Bombyx mori* and *Antheraea mylitta* (Tasar) silk microfibres and microfibre-functionalised *B. mori* hydrogels.

The *B. mori* or *A. mylitta* Tasar silk microfibre syntheses were based on a previously reported technique [13] which was further adapted for the Tasar silk (Figure 1 and Figure S1). Briefly, 1 g of dried, degummed silk fibres were submerged in 15 mL of 17.5 M NaOH solution for 20 min for *B. mori* silk fibres and for 5 h for Tasar silk fibres (Figure S1). The fibres were stirred using a spatula for 12 min. Next, 150 mL of deionised water was added, followed by centrifugation at 48,384× *g* for 20 min. The supernatant was discarded, and the pellet was resuspended in 50 mL of deionised water, stirred and centrifuged again. This step was repeated 5 times. After resuspension of the pellet in deionised water, the pH of the suspension was measured and adjusted to 7.0 by the dropwise addition of HCl. The neutralised silk fibre suspension was centrifuged again for 20 min, and the final silk fibre pellet was resuspended in 2 mL of deionised water. The silk fibres were filtered through a 500 µm strainer to create a major size fraction between 250 and 500 µm. Samples (100 µL) of each silk fibre were lyophilised for concentration measurements. For quantitative fibre length assessments, sample images were captured using light microscopy and analysed using ImageJ for Windows 1.8.0 (National Institutes of Health, Bethesda, Rockville, MI, USA).

### 2.4. Self-Assembling B. mori Silk Hydrogels with and without Microfibres

Self-assembling silk hydrogels were prepared as described previously [29]. Briefly, 4 mL of 3% (*w*/*v*) *B. mori* silk solution was transferred to a 15 mL Falcon tube and sonicated using a digitally controlled probe sonicator (Sonoplus HD 2070, Bandelin, Berlin, Germany) fitted with a 23 cm long sonication tip (0.3 cm diameter tip and tapered over 8 cm) (Figure S1). The sample, while kept on ice, was exposed to 30% amplitude for 4 sonication cycles (one cycle consisted of 30 s on and 30 s off) typically to initiate the solution–gel transition. The chosen silk microfibre type was mixed into the 3% (*w*/*v*) *B. mori* silk solution as the liquid silk underwent the sol–gel transition.

### 2.5. Scanning Electron Microscopy (SEM)

*B. mori* and Tasar silk microfibre suspensions were adjusted to a concentration of 10 mg/mL. A 20 μL suspension of microfibres was pipetted onto a silicon wafer and lyophilised for 24 h at −10 °C and 0.14 mbar. The dried samples were sputter-coated with 15 nm of gold using an ACE200 low-vacuum sputter-coater (Leica Microsystems, Wetzlar, Germany). The fibre samples were imaged using field emission scanning electron microscopy (FE-SEM; SU6600 instrument; Hitachi High Technologies, Krefeld, Germany) with a 5 kV accelerating voltage. The hydrogel samples were imaged with Quanta FEG-ESEM (FEI company, Hillsboro, OR, USA; now part of Thermo Fisher Scientific Inc., Waltham, MA, USA). The images were processed using ImageJ for Windows 1.8.0 (National Institutes of Health, Bethesda, Rockville, MI, USA).

### 2.6. Fourier Transform Infrared Spectroscopy (FTIR)

The secondary structures of the *B. mori* and Tasar silk microfibres and *B. mori* hydrogels were measured as detailed previously [30]. Briefly, all three materials were frozen overnight at −80 °C and lyophilised in a freeze dryer (Epsilon 2-4 LSC, Martin Christ Gefriertrocknungsanlagen GmbH, Osterode am Harz, Germany). The secondary structure of the silk microfibres was analysed using a TENSOR II FTIR spectrometer (Tensor II Bench ATR IR, Bruker Optik GmbH, Ettlingen, Germany). Each sample was exposed to 32 scans for the background and 128 scans for the sample at 4 cm^−1^ resolutions over a wavenumber range of 400–4000 cm^−1^. The secondary structures were assigned as detailed previously [30]. An air-dried film was used as a negative control, and a silk film treated with 70% ethanol was used as a positive control.

### 2.7. Rheology of Silk Hydrogels 

In brief, 2% and 10% (*w*/*v*) concentrations of silk microfibres were prepared in distilled water and sterilised using UV radiation. A 1.8 mL sample of a 3% (*w*/*v*) silk hydrogel was then sonicated using a digitally controlled probe sonicator. The silk microfibre type chosen for the test was added to the 3% (*w*/*v*) silk solution undergoing the solution–gel transition and mixed gently. The sample was transferred to a mould to form a hydrogel 11 mm in diameter, with an average thickness of 9 mm. 

The 3% (*w*/*v*) silk hydrogel microfibre systems were equilibrated overnight in Dulbecco’s Modified Eagle’s Medium (DMEM) containing 1 g/L D-glucose. The hydrogels then underwent rheology characterisation (HAAKE MARS Rheometer, Thermo Fisher Scientific, Loughborough, UK) using a 20 mm diameter plate set to 25 °C and an appropriate gap size. The storage modulus (G’) was measured using a time sweep over a strain of 0.01–100% at a frequency of 1.0 Hz. The stress relaxation rate (G”) was measured at a 15% strain. The stress relaxation was recorded every 10 s for a total of 250 s. Stress was normalised by the initial stress, and the stress relaxation half-time (i.e., the time at which the stress is relaxed to half of the initial stress) was calculated as described previously [31]. 

### 2.8. Cell Assays 

Human iPSC-derived MSCs were purchased from the American Type Culture Collection (Manassas, VA, USA). Briefly, the iPSC-MSCs were cultured in complete culture medium as monolayers in tissue culture plates and on a 3% (*w*/*v*) silk hydrogel composite containing 2% or 10% (*w*/*v*) silk microfibres. Unless otherwise indicated, the cells were seeded at 5000 cells/cm^2^. Cells were cultured at 37 °C in 95% relative humidity and 5% CO_2_. The iPSC-MSCs were used for up to three passages.

Cell viability was measured at 24 h after seeding by adding 25 μL of resazurin (440 μM stock in phosphate-buffered saline, Thermo Fisher Scientific, Waltham, MA, USA). The cells were transferred to an incubator (humidified atmosphere of 5% CO_2_ at 37 °C) for 4 h to allow resazurin metabolism. Then, 100 μL of the supernatant was transferred into a black 96-well plate (Sigma-Aldrich, Merck Life Science UK Limited, Dorset, UK), and the fluorescence was measured using a fluorescence plate reader (POLARstar Omega BMG LABTECH GmbH, Ortenburg, Germany) with a fixed photomultiplier tube (800), with excitation at 560 nm and emission at 590 nm. Blank hydrogels from the same time points were used as controls to subtract background fluorescence, and iPSC-MSCs treated without silk hydrogels were used as a positive control. The iPSC-MSC DNA content was measured as detailed previously [12]. 

### 2.9. Cell Staining and Image Analyses

The iPSC-MSCs were cultured for 24 h on silk hydrogel substrates in glass-bottomed microwell dishes. The hydrogels were then fixed with 4% *v*/*v* methanol-free formaldehyde, permeabilised in 0.1% *v*/*v* Triton-X 100 for 15 min and blocked in 1% (*w*/*v*) bovine serum albumin (BSA; Sigma-Aldrich, Merck Life Science UK Limited, Dorset, UK ) for 1 h at room temperature. For actin filament visualisation, the cells were stained for 1 h with 0.165 μM phalloidin-Alexa488 according to the manufacturer’s instructions (Thermo Fisher Scientific Inc., Waltham, MA, USA) and imaged with an upright epifluorescence microscope (Nikon Eclipse E600). Exposure time and other image settings for each respective channel were held constant during imaging. All images were equally adjusted, processed and analysed in ImageJ 1.8.0 (National Institutes of Health, Bethesda, Rockville, MI, USA). The images were analysed as detailed previously [12]. 

### 2.10. Statistical Analyses and Presentation

BioRender was used to create image diagrams. Data were analysed using GraphPad Prism 10.0.2 (GraphPad Software, La Jolla, CA, USA). One-way analysis of variance (ANOVA), followed by Tukey’s multiple comparison test, was used to compare the viability and attachment of iPSC-MSCs growing on the composite silk microfibre hydrogels versus iPSC-MSCs cultured on control substrates. Statistical significance is indicated by asterisks in each figure legend and assigned as follows: * *p* ≤ 0.05, ** *p* ≤ 0.01 *** *p* ≤ 0.005 and **** *p* ≤ 0.0001. All data are plotted as mean ± standard deviation (SD). The number of experiment repeats (n) is described in each figure legend. 

## 3. Results

**Figure 2:**
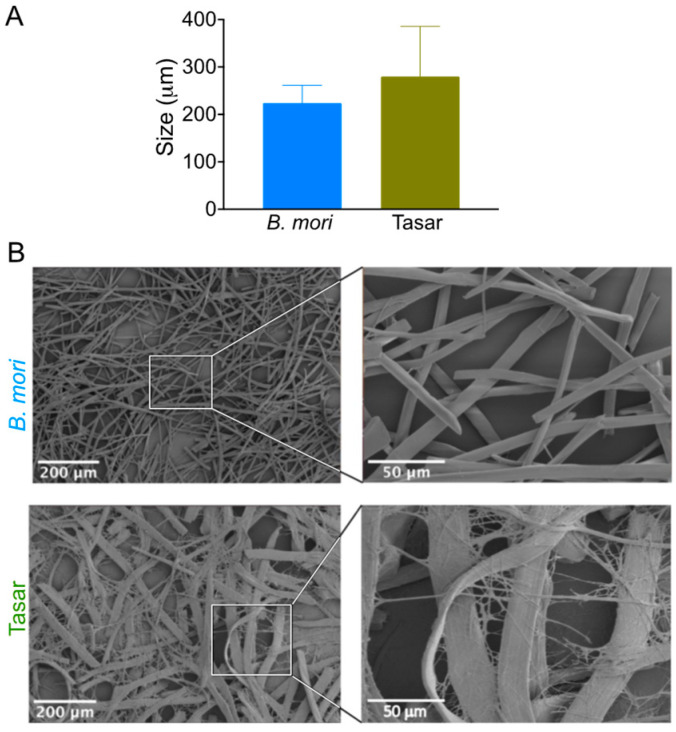
Morphology of silk microfibres. *Bombyx mori* and *Antheraea mylitta* (Tasar) silk microfibre characterisation using (**A**) size measurements (analyses of light microscopy images) and (**B**) surface morphological assessment of silk microfibres with scanning electron microscopy (scale bar, 200 µm; zoom, 50 µm).

The average lengths of *B. mori* and Tasar silk fibres were 223 ± 97 μm and 279.83 ± 105.67, respectively; this difference was not statistically significant (Figure 2A). Qualitative surface morphology assessment indicated that both microfibre types had a similar cylindrical shape, although the diameter of the Tasar silk fibre was approximately twice that of the *B. mori* fibre. The surface of the *B. mori* silk microfibres was generally smooth and glossy, whereas the microfibrils of the Tasar silk microfibres had a loose appearance (Figure 2B).

**Figure 3:**
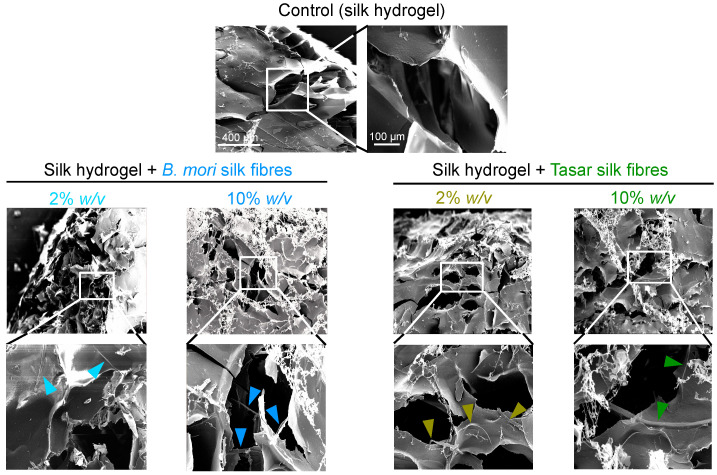
Silk hydrogel composites containing 2% and 10% (*w*/*v*) *Bombyx mori* and *Antheraea mylitta* (Tasar) silk microfibres. The hydrogel morphology was assessed using scanning electron microscopy (SEM) (scale bar, 400 µm; zoom, 100 µm). Silk hydrogels without microfibres were used as controls. Silk microfibres are indicated by arrows.

The morphologies of the control silk hydrogels and those spiked with 2% and 10% (*w*/*v*) silk microfibres were observed using SEM (Figure 3). Control silk hydrogels showed a uniform pore structure (typically 250–450 μm in diameter) and high porosity, while the fibre-reinforced hydrogels typically had uniform but smaller pores (approximately 200–250 μm in diameter) (Figure 3). Both *B. mori* and Tasar silk microfibres were embedded within the silk hydrogel matrix. Increasing the silk microfibre content further reduced the internal porosity of the hydrogels, but the uniform structure was maintained (Figure 3).

**Figure 4:**
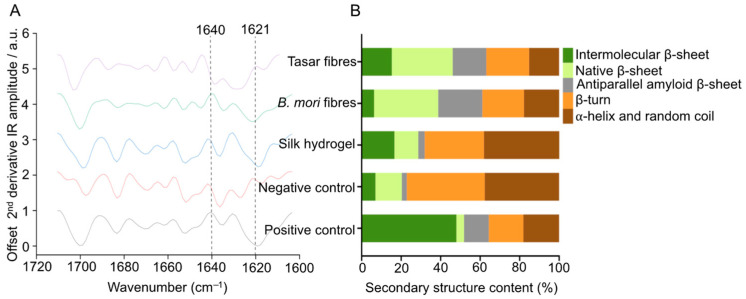
Secondary structure of silk samples. (**A**) Fourier transform infrared (FTIR) spectra and peak assignment. FTIR spectra of *Bombyx mori* and *Antheraea mylitta* (Tasar) silk microfibres, silk hydrogels, an air-dried film (negative control) and a 70% ethanol-treated silk film (positive control). Dashed lines indicate the β-sheet peak (1621 cm^−1^) and the α-helix peak (1640 cm^−1^). (**B**) Quantitative secondary structure analyses of the respective samples. Secondary structures and analysis are as detailed previously [30].

Assessment of the secondary structure of both microfibres and hydrogels using FTIR revealed the typical amide I absorption peak at 1600–1700 cm^−1^ in all silk samples (Figure 4). The spectra of silk films that had been air-dried only (negative control; amorphous) had the lowest β-sheet content (22.86%), while those treated with 70% *v*/*v* ethanol/distilled water (positive control; crystalline) had the highest β-sheet content (64.39%) (Figure 4). The β-sheet contents of Tasar and *B. mori* silk microfibres were 63.13% and 61.12%, respectively (Figure 4). The β-sheet content of the unmodified silk hydrogel was 31.92%. 

**Figure 5:**
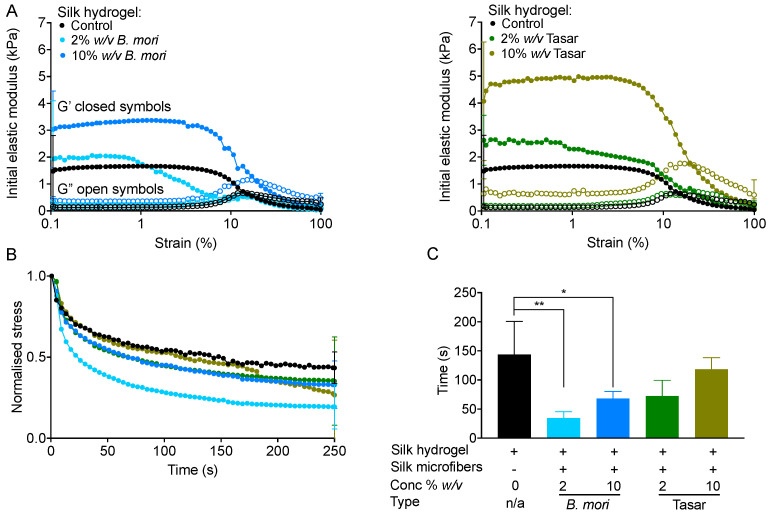
Impact of the microfibre amount and type on silk hydrogel mechanical properties. (**A**) Stiffness, (**B**) stress relaxation time profiles and (**C**) normalised stress relaxation time for 3% (*w*/*v*) self-assembled silk hydrogels (control) with and without microfibres. Data are presented as mean ± SD; n = 3 independent experiments; * *p* ≤ 0.05, ** *p* ≤ 0.01.

Comparison of the mechanics of the 3% (*w*/*v*) silk hydrogel to those of silk hydrogels containing *B. mori* and Tasar silk microfibres (Figure 5A) revealed the lowest stiffness (1.47 kPa) for the silk hydrogels without microfibres (Figure 5A), as well as increasing hydrogel stiffness as the silk microfibre content increased (Figure 5A). Silk hydrogels containing 10% (*w*/*v*) Tasar silk microfibres had the highest stiffness (4.07 kPa), followed by silk hydrogels containing 10% (*w*/*v*) *B. mori* silk microfibres (3.03 kPa), 2% (*w*/*v*) Tasar (2.62 kPa) and then 2% (*w*/*v*) *B. mori* (1.93 kPa) silk microfibres (Figure 5A). Determination of the stress relaxation (Figure 5B) and the stress relaxation half-time (Figure 5C) revealed that the control silk hydrogel with no fibres had the longest stress relaxation time (144 s). However, independent of the fibre type, the fastest stress relaxation was observed with a rank order of 2% (*w*/*v*) > 10% (*w*/*v*) > unmodified hydrogels, and this effect was greatest for *B. mori* fibres (Figure 5C). 

**Figure 6:**
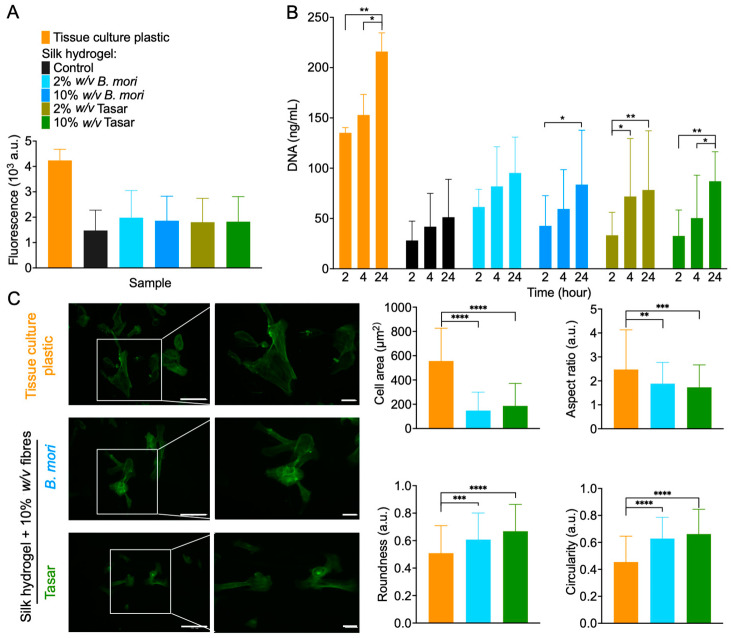
Biological response of human stem cells (iPSC-MSCs) towards two-dimensional silk hydrogel culture substrates. (**A**) Cell metabolic activity at 24 h after seeding on various silk hydrogels or on tissue culture-treated polystyrene (control). (**B**) Quantification of iPSC-MSC cell attachment. Control: iPSC-MSCs seeded on tissue culture-treated plastic were used as control. Data are presented as mean ± SD; n = 3 independent biological experiments. (**C**) Impact of substrate mechanics on iPSC-MSC cytoskeletal organisation. The images show F-actin cytoskeleton staining. All analyses were performed with reference to cell area, aspect ratio, roundness and circularity. Scale bars = 20 and 40 μm. Quantification of the morphological characteristics of iPSC-MSCs (106 cells in n = 21 images from three pooled experiments). One-way analysis of variance (ANOVA), followed by Tukey’s multiple comparison test, was used to compare the silk hydrogel composites with tissue culture-treated plastic; * *p* ≤ 0.05, ** *p* ≤ 0.01 *** *p* ≤ 0.005 and **** *p* ≤ 0.0001.

The responses of iPSC-MSC cells towards these hydrogel substrates were also measured to determine cell attachment and metabolic activity (Figure 6B and Figure 6A, respectively). Assessment of metabolic activity showed no statistically significant differences between iPSC-MSCs cultured on 2D silk hydrogels containing *B. mori* and Tasar silk microfibres. However, the metabolic activity of cells grown on tissue culture-treated polystyrene had twice the values observed for hydrogel substrates (Figure 6A). 

Here, the DNA content was used to quantify the degree of cell attachment. All hydrogel substrates showed a time-dependent increase in cell attachment. The iPSC-MSCs grown on 2% (*w*/*v*) *B. mori* microfibre hydrogels showed substantial increases in cell numbers at 2 h after seeding. Compared to the cell adhesion to the silk hydrogel without fibres, cell adhesion was almost doubled for all reinforced silk hydrogel substrates at 24 h. Surprisingly, no statistically significant differences were noted for cell adhesion to the silk hydrogels containing *B. mori* and Tasar silk microfibres (Figure 6A).

Assessment of the cell morphology at the 24 h culture time point revealed that the iPSC-MSC positive controls cultured on tissue culture-treated polystyrene showed greater stretched and elongated morphology when compared to that of cells grown on silk hydrogels containing either 2 or 10% (*w*/*v*) *B. mori* or Tasar silk microfibres (Figure 6C). The iPSC-MSCs cultured on tissue culture-treated polystyrene showed signs of membrane protrusion, with local actin polymerisation, whereas these features were infrequently observed in the iPSC-MSCs cultured on silk hydrogels or their respective silk microfibre hydrogels (Figure 6C). Quantifications of the cell area, aspect ratio, roundness and circularity revealed a greater roundness and circularity for iPSC-MSCs cultured on reinforced silk hydrogels than on tissue culture plastic.

## 4. Discussion

Reinforcing hydrogels with silk microfibres broadens their use in biomedical and engineering applications. For example, fibre-reinforced hydrogels have increased mechanical strength, making them useful for hard tissue engineering applications [14,15,32]. This ability to tune the hydrogel mechanical properties is critical as it enables matching of the construct to a wider set of target tissues (which can have a broad spectrum of mechanics). This mechanical matching, in turn, is important because it promotes better integration and, ultimately, better functional restoration. Substrate stress relaxation is also important as it has known effects on cell biology, including the functioning of stem cells [33]. However, the study of stress relaxation in the context of silk is only now being addressed [12], and we still do not know how the addition of fibres may impact stress relaxation. The added microfibres can also introduce topographical cues that can guide cell adhesion, alignment and migration [14,15,32]. 

In the present study, we included silk microfibres to improve the weak mechanical properties of sonication-induced silk hydrogels. To the best of our knowledge, this study is the first to develop Tasar fibre-reinforced *B. mori* silk hydrogels and to examine their substrate mechanics, including viscoelasticity. Tasar silk fibres are particularly attractive in tissue engineering because their primary structure contains seven RGD sequences per silk molecule that can be exploited to guide cell attachment [20]. Here, we tested both *B. mori* and Tasar silk microfibres as silk hydrogel reinforcements and compared the resulting hydrogels to a control silk hydrogel without microfibres. 

We first used the protocol described by Mandal and co-workers to generate silk microfibres [13]. Our aim was to generate microfibres that were 200 to 400 μm in length because this length has previously shown the best performance in silk scaffolds [13] and hydrogels [14]. We used light microscopy to assess the fibre lengths, and our *B. mori* data (Figure 2) are comparable to those reported in previous studies (e.g. [14,34]). We then used the same parameter space for Tasar silk, but we extended the incubation time to 5 h to yield microfibres of comparable length to the *B. mori* fibres. Electron microscopy examination of the fine surface structure of the fibres revealed that our Tasar silk microfibres were approximately twice as wide as the *B. mori* fibres (Figure 2B). This difference arises due to the different spinning geometries inherent in *B. mori* and Tasar silkworm physiology. The processed *B. mori* silk microfibre had a smooth surface appearance, whereas the Tasar silk microfibres showed some microfibrils, as reported previously [35]. Tasar silk cocoons are tightly woven to provide maximum protection during the four-month pupal diapause of the silkworm [35]. By contrast, the cocoon of the fully domesticated *B. mori* cocoon is readily reeled and the pupa immediately starts metamorphosis. 

We also determined the fibres’ secondary structure using FTIR spectroscopy. One factor that impacts the structure of a silk microfibre is its beta-sheet content, which forms via hydrogen-bonding interactions between the silk chains. This hydrogen bonding creates a highly ordered and rigid crystalline structure [36]; therefore, the presence of beta-sheets imparts greater stiffness and strength to the silk material because the hydrogen bonds in the beta-sheet structure resist deformation and provide mechanical support [36]. The secondary structures of Tasar and *B. mori* microfibres, as well as that of the 3% (*w*/*v*) silk hydrogels, were analysed and deconvoluted between wavenumbers 1600 and 1710 cm^−1^ (amide I band) (Figure 4). The findings showed that the beta-sheet contents of the microfibres of both Tasar and *B. mori* silks were significantly higher than the beta-sheet content of the silk hydrogels or the air-dried silk films (negative control) (Figure 4). These results were expected and are comparable to data reported previously (e.g., [12,37]).

Silk hydrogels, especially those containing >3% (*w*/*v*) silk, are prone to cracking under low-stress conditions due to a lack of energy dissipation mechanisms [38]. The addition of silk microfibres to this type of brittle hydrogel matrix increases the hydrogel stiffness, resulting in improved mechanical properties [14,15,32]. A spectrum of fibre-reinforced hydrogels has been investigated for tissue engineering by incorporating *B. mori* fibres within the hydrogel matrices [14,15,32]. For example, adding 2% (*w*/*v*) *B. mori* silk microfibres to an 8% (*w*/*v*) silk hydrogel created a structure that mimicked the mechanical properties of native cartilage in vitro [14]. In the present study, silk hydrogels were reinforced with silk microfibres using the same (i.e., 2% [*w*/*v*]) and a higher (i.e., 10% [*w*/*v*]) concentration of fibres to assess the impact of silk microfibres on the mechanical properties of the silk hydrogel. Increasing the concentration of silk fibres increased the initial modulus (stiffness) (Figure 5A,B) in agreement with previous findings [14,15]. We have previously shown that 4% (*w*/*v*) silk hydrogels show stress relaxation [12]; however, the impact of fibre reinforcement is unknown. 

Microfibre reinforcement increases the mechanical strength of hydrogels. However, in the present study, we now provide the first demonstration that fibre addition also increases the speed of stress relaxation, thereby enabling greater material plasticity. We speculate that the presence of microfibres provided ‘flow points’ within our hydrogels. Therefore, the application of stress eases the material flow, resulting in shorter material relaxation times. The ‘flow points’ are more effective when provided by the smooth *B. mori* microfibres than by the coarse Tasar microfibres. However, our experimental data also showed that the increased speed of stress relaxation was inversely correlated with the fibre content. This observation may seem counterintuitive, but it might be explained by changes in the hydrogel morphology. Our morphological analyses revealed that structural uniformity was maintained in the microfibre-reinforced hydrogels, but the presence of silk microfibres reduced the internal porosity in a manner that correlated with the fibre content (Figure 3). Therefore, we speculate that the balance between ‘flow points’ and internal porosity governs the stress relaxation time (Figure 5B,C). 

We also assessed the impact of silk hydrogel reinforcement with *B. mori* and Tasar silk microfibres on iPSC-MSC biology by monitoring cell attachment over the first 24 h after seeding (Figure 6). All hydrogels strengthened with *B. mori* and Tasar silk microfibres showed improved iPSC-MSC attachment. In particular, hydrogels reinforced with Tasar silk microfibres at either 2% or 10% (*w*/*v*) exhibited substantially greater cell attachment compared to *B. mori* fibre-reinforced or control hydrogels (Figure 6). Improvement in cell attachment in response to Tasar silk is therefore in line with previous findings using films and three-dimensional scaffolds (e.g., [21]). However, we currently do not know how the loosely appearing Tasar microfibrillar networks (Figure 2B) impact cellular responses. One might speculate that the increased surface area contributes to improved cell attachment. Notably, an assessment of cell metabolic activity at 24 h post-seeding revealed values that were approximately 50% lower in cells cultured on reinforced hydrogels than in control cells cultured on tissue culture polystyrene (which essentially enables 100% cell attachment). Therefore, our data not only corroborate the cell attachment data, but they also highlight that further work is needed to maximise cell–substrate engagement. 

Finally, morphological assessment of iPSC-MSCs grown on silk hydrogels reinforced with 10% (*w*/*v*) microfibres revealed that the iPSC-MSCs were able to stretch and elongate on these substrates. However, the iPSC-MSCs grown on tissue culture plastic substrates had the largest surface area (i.e., spreading), as is typical and expected for cell growth on rigid substrates. Classical cell geometry studies have demonstrated that cell shape directly impacts cell biology, including proliferation [39], apoptosis [40] and differentiation [41]. Our understanding of the underlying molecular biology of this substrate–cell ‘signalling synapse’ is now emerging. For example, cell geometry has been shown to influence nanostructure and lipid assembly within the cell plasma membrane by triggering signalling events [42]. We, therefore, speculate that the different iPSC-MSC geometries observed here will also have impacts on cell metabolism and ultimately on cell differentiation.

## 5. Conclusions

We have examined the impact of silk hydrogel reinforcement on substrate mechanics and biology in iPSC-MSCs. Here, *B. mori* and Tasar silk microfibres were successfully manufactured and embedded in *B. mori* silk hydrogels, with a resulting improvement in the mechanical strength and the rapidity of stress relaxation of the hydrogels. When compared to control silk hydrogels, silk hydrogels functionalised with silk microfibres promoted short-term iPSC-MSC adhesion and metabolic activity. This work has the potential to open up the wider use of silk fibroin for the creation of structured hydrogels, especially by blending different silk types and material formats. Overall, the findings of this study demonstrate that the reinforcement of silk hydrogels with different concentrations of silk microfibres alters the hydrogels’ mechanical properties and impacts iPSC-MSC cell biology.

## Data Availability

Raw data are available upon request.

## Supplementary Materials

The following supporting information can be downloaded at: https://www.mdpi.com/article/10.3390/cells13010010/s1, Figure S1: Diagram depicting the manufacture of *Bombyx mori* and *Antheraea mylitta* (Tasar) silk microfibres and *B. mori* hydrogels.
