# Supplementary material for: Microfibre-Functionalised Silk Hydrogels
Source: Cells. 2023 Dec 20;13(1):10. doi: 10.3390/cells13010010 (PMC10777932; doi:10.3390/cells13010010)
Supplement: Supplementary file 1 [file cells-13-00010-s001.zip › cells-2748861-supplementary.pdf]

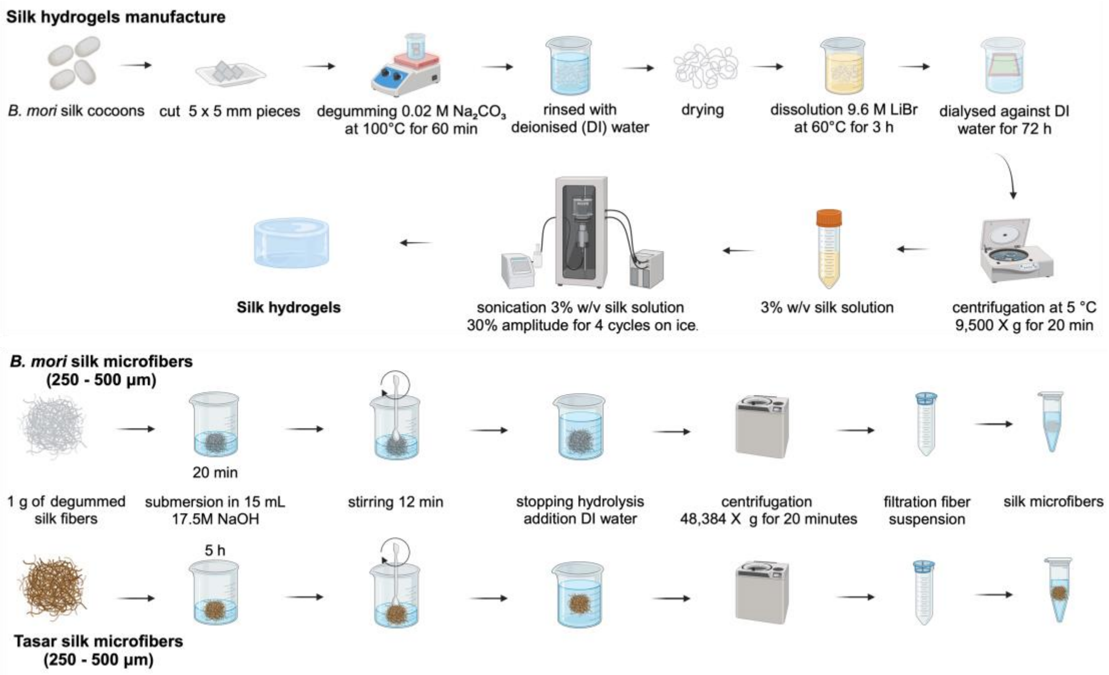


**Figure S1.** Diagram depicting the manufacture of *Bombyx mori* and *Antheraea mylitta* (Tasar) silk microfibres and *B. mori* hydrogels**.**
